# CFD-DEM Coupling Model for Deposition Process Analysis of Ultrafine Particles in a Micro Impinging Flow Field

**DOI:** 10.3390/mi13071110

**Published:** 2022-07-15

**Authors:** Yanru Wang, Zhaoqin Yin, Fubing Bao, Jiaxin Shen

**Affiliations:** Zhejiang Provincial Key Laboratory of Flow Measurement Technology, China Jiliang University, Hangzhou 310018, China; s20020804055@cjlu.edu.cn (Y.W.); dingobao@cjlu.edu.cn (F.B.); p20020854067@cjlu.edu.cn (J.S.)

**Keywords:** ultrafine particle, impaction, CFD-DEM, deposition characteristic

## Abstract

Gas with ultrafine particle impaction on a solid surface is a unique case of curvilinear motion that can be widely used for the devices of surface coatings or instruments for particle size measurement. In this work, the Eulerian–Lagrangian method was applied to calculate the motion of microparticles in a micro impinging flow field with consideration of the interactions between particle to particle, particle to wall, and particle to fluid. The coupling computational fluid dynamics (CFD) with the discrete element method (DEM) was employed to investigate the different deposition patterns of microparticles. The vortex structure and two types of particle deposits (“halo” and “ring”) have been discussed. The particle deposition characteristics are affected both by the flow Reynolds number (*Re*) and Stokes number (*stk*). Moreover, two particle deposition patterns have been categorized in terms of *Re* and *stk*. Finally, the characteristics and mechanism of particle deposits have been analyzed using the particle inertia, the process of impinging (particle rebound or no rebound), vortical structures, and the kinetic energy conversion in two-phase flow, etc.

## 1. Introduction

The ultrafine particle deposition process in a micro impinging flow field widely exists in industry engineering of multiphase flow. The typical device is composed of one or more nozzles and an impaction plate (collection plate) below the nozzle for ultrafine particle acceleration, collision on the wall, then sedimentation [1]. When the particle-laden jet passes through the nozzle, the impaction plate changes the original direction of the flow, thus it makes a turn above the plate. Particles with large inertia will impinge onto the plate surface to be collected for further application. Particle deposition pattern and collection efficiency are key characteristics to evaluate the process of ultrafine particle deposition on the surface. The unwanted particle deposition can adversely affect miniature systems, such as cold spray, vacuum evaporation, and inertial impactor.

Recently, scholars have launched a series of studies on how to improve the performance of the impactor. Lee et al. [2] proved that the rebound effect of particles can be reduced for high collection efficiency by using oiled substrates through the experiment. Arffman et al. [3] referred the impact of turbulence on impactor resolution needs to be considered when the flow Reynolds number is over 1800. Interestingly, they noted that particles did not necessarily deposit in the center of the plate below the nozzle. Actually, in the early time, Sethi and John [4] reported that ring-shaped particle deposits formed on the collection plate when the particle size is 3 µm. Recently, it has been stated that the thickness and diameter of the ring deposition changed with the ratio of the nozzle-to-plate distance to the nozzle diameter and the particle size through experiments [5]. Soysal et al. [6] analyzed particle deposition patterns with a lab-made inertial impactor. They defined the deposit shape for three types, namely the ring, the halo, and the disk. The particle number was reduced in the primary impaction zone with the increasing particle size due to the bounce effect. García-Ruiz et al. [7] thought the formation of halo deposits was related to particle inertia and gravity. However, the halo deposits should be avoided in the impactor, if possible, since the collection of particles in the “halo” relies on the size of the collection plate. Hence, the particle collection zone should be small enough. Furthermore, when the influence of the halo deposit is considered, the collection efficiency becomes low [8]. The “halo” is regarded as an intractable problem. Therefore, it is urgent to study the cause of the formation of these deposits.

Several mechanisms have been proposed through analysis of experiment results to explain the formation of the “halo” deposits, such as gravitational sedimentation, the hydrodynamic effect, particle inertia, Magnus lift, Saffman lift, and particle rebound and re-entrainment in most researches [6,7,8,9,10,11]. The combined method of computational fluid dynamics and discrete particle method (CFD-DPM) considered a powerful and reliable technique was utilized to simulate particle movement in the impactor to achieve the location of particle deposition [12]. In order to simplify the problem and reduce calculation costs, the complex particle–particle and particle–fluid interactions have been ignored in previous studies. However, Wu et al. [13] noted the particle collision is obvious when the discrete phase volume fraction is larger than 0.008% in an impinging jet. Trofa et al. [14] observed that 5 µm particles will modify the surrounding flow, affecting particle movement when particle volume fraction is about 0.05–0.1%. Hence, particles can affect fluid motion in microchannels. When the particle volume fraction is between 10^−6^–10^−3^, a two-way coupling algorithm of particle–fluid and particle–particle interactions is needed, such as the combined approach of computational fluid dynamics and the discrete element method (CFD-DEM) [15]. The DEM method based on soft-sphere model can accurately calculate the trajectory of each particle and it takes the particle–particle collision and particle–wall collision into account. Particles colliding are allowed to deform, so that elastic/plastic and frictional forces can be calculated in this approach. Although the computational time is long, which limits the particle size and particle number, parallel processing capability is an advantage of the DEM method to scale up the particle number [16]. CFD-DEM approach has been applied to simulate particle behavior in the impactor [17]. Ohsaki et al. [18] simulated the behavior of fluid and particles in the cascade impactor using the CFD-DEM method with Johnson–Kendall–Roberts (JKR) theory [19]. They noted particles would reach the lower stages without considering adhesion force, indicating that both inertia and adhesion forces affect particle deposition. They also proved that the coupling CFD-DEM method can correctly calculate the particle motion in the cascade impactor through experiments. Flynn et al. [20] found the impact of cross–flow interactions will increase at high flow velocity when operating the Andersen Cascade Impactor (ACI), resulting in disproportionate particle collection. High wall losses attributed to the large recirculation vortices occurred. Ponzini et al. [21] presented the results of numerical and experimental studies on the aerosolization process for a dry powder inhaler (DPI). It might help people to better understand not only the particle behavior but also the disaggregation/aerosolization in the DPI.

Therefore, in order to qualify the influence of ultrafine particles deposition characteristics in the micro impinging flow field, the CFD-DEM coupling model based on the local volume average method has been used to solve the movement of fluid flow and ultrafine spherical particles in this paper. The results will be important for optimizing the inertia impactor performance and improving the technologies such as surface coatings, microparticle measurement, and drug delivery, etc. [22,23,24,25].

## 2. Model Formulation

### 2.1. The Governing Equations of the Fluid Phase

The following assumptions were used for the continuous phase. On one hand, the flow is laminar [26], and there is no slip between particles and the wall surface. On the other hand, the flow is assumed to be incompressible when the Mach number is smaller than 0.3. The fluid phase is governed by the continuity and Navier–Stokes (N–S) equations written as:

(1) $$\frac{\partial \rho }{\partial t}+\nabla \cdot (\rho u)=0$$

(2) $$\frac{\partial \left(\rho u\right)}{\partial t}+\nabla \cdot (\rho uu)=-\nabla p+\nabla \cdot (\tau )+\rho g-S_{ai}$$

where *ρ*, *u*, *p* and *τ* describe the density, velocity, pressure, and viscous stress tensor of gas. *g* is the gravitational acceleration. *S_ai_* represents momentum transfer term between the fluid and the *i*th particle phase.

(3) $$S_{ai}=\frac{\sum_{i}^{n}F_{a,i}}{V_{cell}}$$

where *F*_*a*,*i*_ and *V_cell_* are the total force exerted on the *i*th particle and volume of the cell, respectively.

### 2.2. The Motion Equation of Particles in the Flow

Particle tracking is conducted by considering various forces in a Lagrangian reference frame. As particles were allowed to rotate also the change of particle angular velocity was calculated. Therefore, the motion equation for each microparticle was solved according to Newton’s second laws [27].

(4) $$m_{p}\frac{du_{p}}{dt}=F_{D}+m_{p}\frac{g\left(\rho_{p}-\rho \right)}{\rho_{p}}+F_{c}$$

(5) $$I_{p}\frac{d\omega_{p}}{dt}=\sum \left(r_{c}\times F_{c}\right)$$

where *m_p_*, *u_p_*, *F_D_*, *F_c_*, *I_p_*, *ω_p_*, *r_c_* indicate the particle mass, particle velocity, drag force, contact force acted on the particle, rotational inertia of the particle, angular velocity of the particle, and distance from the contact point to particle center, respectively. The drag force plays a significant role which is not negligible in any case defined as:

(6) $$F_{D}=\frac{3\mu C_{D}Re_{p}}{4d_{p}^{2}\rho_{p}}(u-u_{p})$$

where *µ*, *ρ_p_*, and *d_p_* are fluid dynamic viscosity, particle density, and particle size, respectively. *Re_p_* and *C_D_* are particle Reynolds number and the drag coefficient, respectively, and they are expressed as:

(7) $$Re_{p}=\frac{\rho d_{p}\left|u-u_{p}\right|}{\mu }$$

(8) $$C_{D}=\{\begin{array}{l} 0.6167+\frac{46.5}{Re_{p}}-\frac{116.67}{{{Re}_{p}}^{2}}(10<Re_{p}<100)\\ 1.222+\frac{29.1667}{{Re}_{p}}-\frac{3.8889}{{{Re}_{p}}^{2}}(1<Re_{p}<10) \end{array}$$

where Equation (8) were based on Morsi and Alexander [28] with spherical particles.

#### 2.2.1. Particle–Particle Interactions

In the DEM calculation, the Hertz–Mindlin model which is used to calculate the basic contact forces was chosen for particle–particle interaction. The contact force *F_c_* is divided into tangential component *F_ct_* and normal component *F_cn_*. *F_ct_* consists of the elastic force *F_et_* and damping force *F_dt_*. *F_cn_* also includes the elastic force *F_en_* and damping force *F_dn_*. *F_et_*, *F_dt_*, *F_en_*, and *F_dn_* are applied according to the Hertz–Mindlin model [29]. In the equations, the normal and tangential components of contact forces depend on the coefficient of restitution and the static and rolling friction coefficients between two particles in contact, as mentioned in [30].

(9) $$F_{c}=F_{ct}+F_{cn}$$

(10) $$F_{ct}=F_{et}+F_{dt}$$

(11) $$F_{cn}=F_{en}+F_{dn}$$

#### 2.2.2. Particle–Wall Interactions

Particle bounce is a major concern when using impactors [5,6,11,31,32,33]. The JKR model has been used to analyze the particle movement in the impactor [18,34,35]. In this paper, Hertz–Mindlin with JKR model was also selected for the contact force of particle-wall interactions [18]. In contrast to the Hertz–Mindlin model, the contact force in the normal direction *F_cn_* contains the damping force *F_dn_* and elastic force *F_JKR_* based on JKR theory. *F_JKR_* accounts for van der Waals forces exerting a great impact within the contact zone between particles and wall. The rebound effect of particles will be reduced using this contact model, thus more particles will be collected to increase the collection efficiency.

(12) $$F_{cn}=F_{JKR}+F_{dn}$$

The normal elastic force *F_JKR_* which represent the general adhesion phenomenon in the contact area is described as [19]:

(13) $$F_{JKR}=-4\sqrt{\pi \gamma E^{\prime }}a^{3/2}+\frac{4E^{\prime }}{3R^{\prime }}a^{3}$$

where *γ*, $E^{\prime }$, $R^{\prime }$, and *a* are the surface energy, equivalent Young’s modulus, equivalent radius of the particle, and the contact half-width, respectively. For the case without adhesion forces, *γ* = 0, *F_JKR_* turns into Hertz normal contact force *F_en_*. $E^{\prime }$ and $R^{\prime }$ are described as:

(14) $$\frac{1}{R^{\prime }}=\frac{1}{R_{i}}+\frac{1}{R_{j}}$$

(15) $$\frac{1}{E^{\prime }}=\frac{\left(1-\nu_{i}^{2}\right)}{E_{i}}+\frac{\left(1-\nu_{j}^{2}\right)}{E_{j}}$$

where *R*, *E*, and *ν* are the particle radius, Young’s modulus, and Poisson’s ratio, respectively. *i* and *j* represent the different particles. Moreover, the normal overlap *δ_n_* between particles is written as follows:

(16) $$\delta_{n}=\frac{a^{2}}{R^{\prime }}\left(1-\sqrt{\frac{4\pi \gamma {R^{\prime }}^{2}}{E^{\prime }a^{3}}}\right)$$

## 3. Numerical Procedure

**Figure 1:**
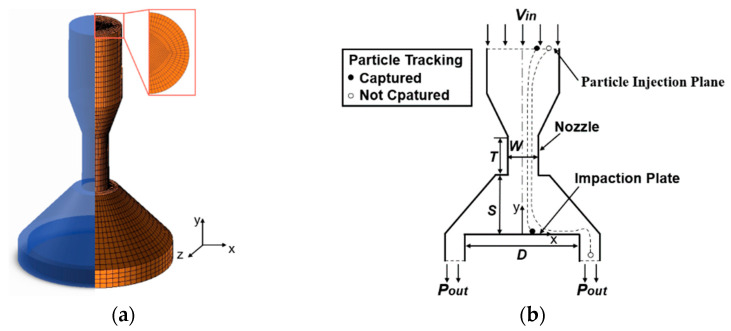
Schematic of the model and the grid model. (**a**) three-dimensional view. (**b**) cross-section view.

The model selected in this paper is based on the shape parameters of the electrical low-pressure impactor (ELPI) in the experiment [34] and the numerical study of a two-dimensional model [26]. In this study, a three-dimensional model was calculated to focus on the axial and radial changes of the flow field and particle movement. The schematic of the model and grid model are shown in Figure 1. Hexahedral structure meshes with the O-block method are conducted in the grid model and local grid refinement is carried out in the jet region. *W* is the round-nozzle diameter, *T* is the length of the nozzle throat, *S* represents the distance from the nozzle to the impaction plate, and *D* represents the diameter of the impaction plate. The model parameters were set at *W* = 1.4 mm, *S* = 3 mm, *T* = 3 mm, and *D* = 7 mm for numerical analysis. Temperatures at the walls and inlet were set to be the same as the ambient temperature 293 K, and all simulations were under the adiabatic condition. The Stokes number (*stk*) is a significant parameter that governs the collection efficiency of inertial impactors and is defined as [35]:

(17) $$stk=\frac{\rho_{p}d_{p}^{2}C_{c}u}{9\mu W}$$

where *C_c_* is the slip correction factor.

CFD method based on the finite volume method was employed to simulate the fluid dynamics. Pressure-based solver and the SIMPLE algorithm were adopted. The flow variable unit centered on the boundary of the grid was reconstructed with 2nd order of accuracy. The second order upwind algorithm was used to solve the momentum. The gas–solid two-phase flow was simulated using a time-accurate transient calculation. The velocity inlet condition at the inlet and pressure outlet condition at the outlet were set as the boundary conditions. The no-slip condition is set for walls.

Furthermore, the convergence criterion for iteratively solving continuity and momentum equations was set at 10^−7^. The gas flow state was in laminar flow with Reynolds number *Re* = *ρuW*/*μ* = 200–1600 [3]. Gravity was enabled due to the particle collection efficiency greatly affected by gravity when the flow Reynolds number is less than 1500 [36]. In the stationary flow field, after convergence was reached, particles were randomly released with the same flow velocity at the inlet and the particle volume fraction was roughly 0.008–0.09%. Particles escaped from the outlet boundary. The particle time step for DEM simulation driven by the Rayleigh times was set at 2 × 10^−8^ s, which is approximately in the order of 9–30% of the Rayleigh time step [37]. The time step of the fluid calculation is an integral multiple of the particle time step [38,39]. Detailed calculation conditions for the CFD-DEM simulation are summarized in Table 1. The numerical simulations were carried out using ANSYS FLUENT15.0 and EDEM2.7 software on an Intel Xeon Platinum 8260 processor with 288 cores and 831 GB of RAM.

## 4. Validation of Solvers

**Figure 2:**
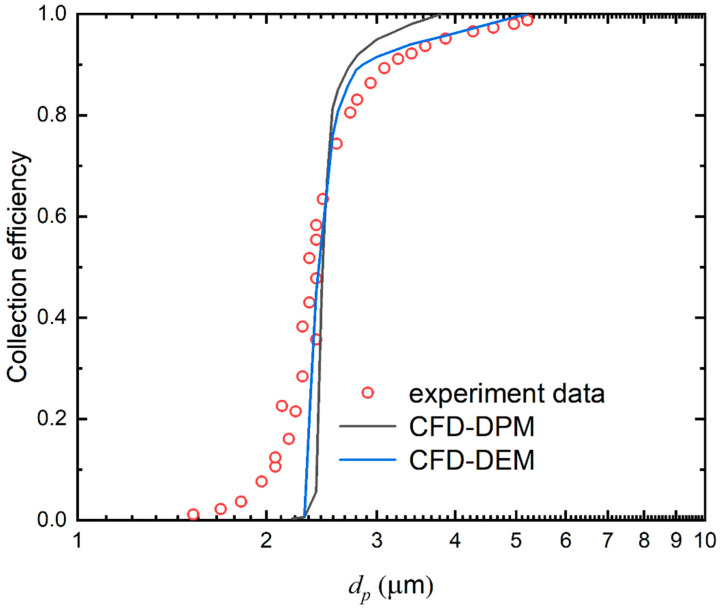
The collection efficiency obtained by the simulated results from CFD-DPM method and CFD-DEM method and experiment data [34].

The grid-independent solution is achieved through a series of computations for four different grid sizes of 450,000, 550,000, 650,000, and 750,000 in Table 2. Generally, it is better to make the length of the CFD mesh element to particle diameter ratio equal to 3. Comparing the average jet velocity through the nozzle under these numbers of grids with the calculated jet velocity, it is found that when cells contain more than 650,000, the deviation is less than 0.66%. 650,000 grids are determined to be sufficient for the present simulation comprehensively considering the computational cost and precision. To evaluate the accuracy of the CFD-DEM approach, the comparison of collection efficiency of particles with a certain size range between experimental data from Marjamäki et al. [34] and the CFD-DPM and CFD-DEM predicted results is illustrated in Figure 2. We assumed that particles are caught when they hit the plate surface in the CFD-DPM method or particle velocity is below 1 × 10^−6^ m/s on the collection plate for the CFD-DEM approach. The curve of collection efficiency through the CFD-DPM method is steeper perhaps by the mass point assumption. In addition, the slopes of the efficiency curves are listed in Table 3. It can be noted that there is a smaller relative error in predicting the slope of the collection efficiency curve by the CFD-DEM coupling model than that of the CFD-DPM model. Consequently, this approach can correctly calculate the flow field and particle behavior.

## 5. Results and Discussions

### 5.1. Particles Deposit Characteristics and Fluid Flow Structure

**Figure 3:**
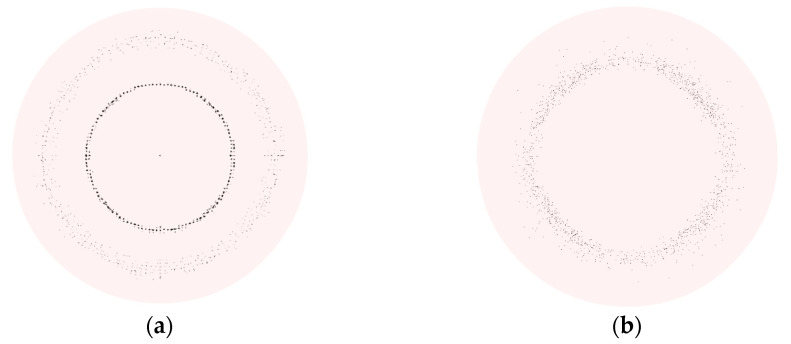
The deposition of spherical particles on the impaction plate. (**a**) “halo” for *Re* = 400, *stk* = 0.25. (**b**) “ring” for *Re* = 700, *stk* = 0.25.

Figure 3 shows the magnitude and shape of the particle deposition on the plate under *stk* = 0.25 for *Re* = 400 and *Re* = 700. Two types of deposits, named as “ring” and “halo” are observed on the plate. For the case of *Re* = 400 and *stk* = 0.25, the particle deposition pattern includes two parts, one appears as the ring-like deposition (primary deposition) away from the stagnation point, and the other is defined as a halo distributed around the primary deposition in Figure 3a. However, it changes to be a “ring” when *Re* = 700 for *stk* = 0.25 in Figure 3b. Similarly, there are the primary ring and another ring in the previous research [7,10]. Halo deposit which is ring-shaped was also mentioned in the experimental results [8,11]. Moreover, the deposition pattern is highly similar to the phenomenon seen in the experimental testing [6] with *d_p_* = 0.92 µm and *d_p_* = 2.07 µm, but the ring deposit occurred with *d_p_* = 2.53 µm and *d_p_* = 3.83 µm on Si surfaces by inertial impaction.

**Figure 4:**
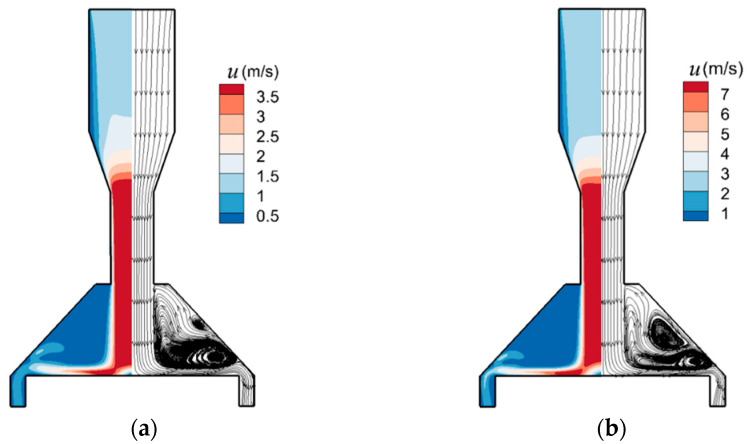
The fluid velocity and streamline of cross-section view of the model. (**a**) *Re* = 400, *stk* = 0.25. (**b**) *Re* = 700, *stk* = 0.25.

The particle behavior is affected by the flow field characteristics. The fluid velocity and streamline in the axial direction (i.e., *y* direction) are shown in Figure 4 under *stk* = 0.25 for *Re* = 400 and *Re* = 700. The maximum flow velocity is at the minimum cross-sectional area. A stagnation region is formed in the center of the impingement zone when a jet impinges vertically on a flat plate. The stagnation region is characterized by high static pressure and decelerated airflow owing to a strong shear at the boundary and the entrainment of the surrounding fluid, hence the fluid velocity of the stagnation region diminishes fast. Then the jet deflects into the radial direction together with the maximum streamline curvature around the jet deflection region [40]. The flow soon comes to the radially expanding wall region, followed by the fluid slowing down by radial spreading. Vortices are initiated by the presence of the impaction plate and sidewall which change the flow direction, and frictional drag. Figure 4 shows two vortices with the clockwise vortex in the upper area and the counterclockwise vortex in the lower area near the radially expanding wall region. As *Re* increases, vortex in the upper area gets larger, while vortex in the lower area gets smaller, followed by the probability of “halo” reducing. Moreover, the vortex core in the lower area moves along the flow direction, resulting in the halo deposit migrating toward the plate edge with the increasing *Re*.

**Figure 5:**
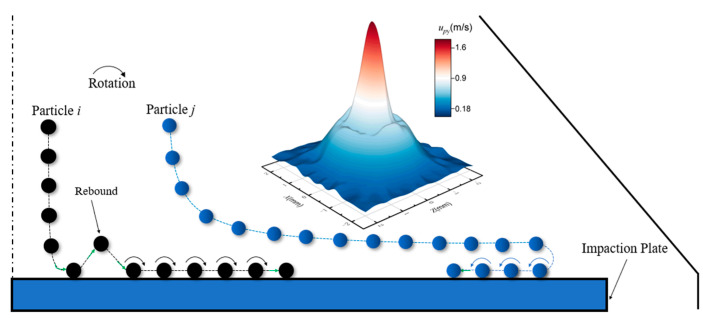
The schematic of particle trajectory above the impaction plate for “halo”.

**Figure 6:**
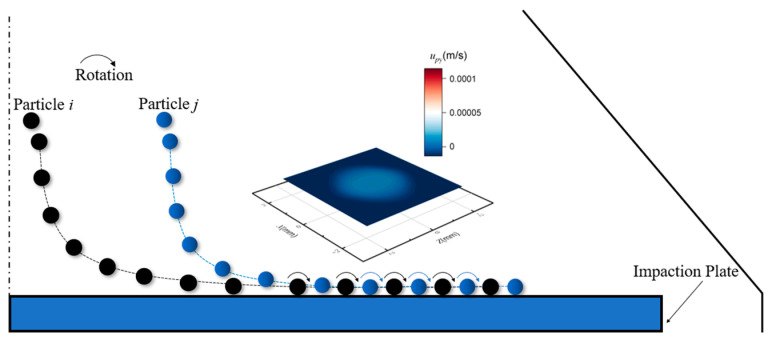
The schematic of particle trajectory above the impaction plate for “ring”.

The location of particles deposited is related to the direction of the vortex structure. To explain the formation of the “halo” and “ring” deposit, Figure 5 and Figure 6 depict schematics of the particle trajectory above the plate under the same *Re* and different *stk* (*stk* is larger in Figure 5). Particles settle out of the impinging jet flows and collide with the wall. *u_py_* represents the velocity magnitude of particles in the *y* direction after the first particle-wall collision. The rebound phenomenon occurs in Figure 5, while almost no rebound occurs in Figure 6 because *u_py_* is especially small. It can be inferred that particles for larger *stk* deviate from the streamlines much sooner and tend to rebound owing to their large inertia. After the rebound, particles are decelerated in the stagnation region and collide with the wall again. They will experience a velocity gradient, with their upper edges being pushed forward by the spreading flow and their lower edges being slowed down by the stationary boundary layer, causing them to roll on the plate surface, then the primary deposit forms. The particle collection area is significantly dispersed from the jet center owing the rebound and flow spreading. However, far from the stagnation region, some particles follow the flow due to the large velocity of air spreading from the plate center, then they become held in the large recirculation regions and move inward. Particles escaping from the initial impaction are collected in the halo deposit attributed to vortices. The primary deposit and halo deposit can be distinguished due to the deceleration of stagnation region and the energy loss owing to the particle-wall collision. For Figure 6, particles with lower *stk* show stronger airflow followability as a result of low inertia, forming the ring deposition. The result demonstrates that the “halo” forms for particles with bigger Stokes number under the same flow Reynolds number.

**Figure 7:**
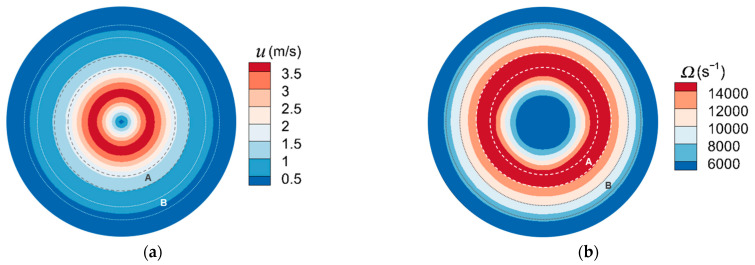
The (**a**) fluid velocity distribution and (**b**) vorticity above the plate for *Re* = 400, *stk* = 0.25. Regions A and B represent the primary deposit and halo, respectively.

**Figure 8:**
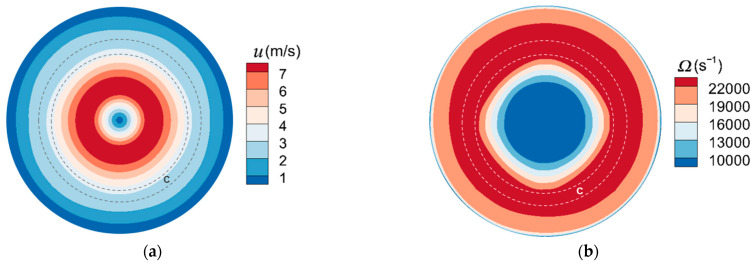
The (**a**) fluid velocity distribution and (**b**) vorticity above the plate for *Re* = 700, *stk* = 0.25. Region C represents the ring deposit.

The fluid velocity and vorticity (*Ω*) above the plate are presented in Figure 7 and Figure 8 under *stk* = 0.25 for *Re* = 400 and *Re* = 700 with the location of “ring” and “halo” marked. The primary deposit and halo are expressed as regions A and B, respectively. The ring deposit is expressed as region C. Result suggests that these depositions are mostly located in the area with low fluid velocity. Particles are subjected to four forces, which are the friction force and adhesion of the plate on particles, the pushing force of the fluid spreading from the core region of the jet to the plate edge, and the particle gravity. They will be deposited when particle kinetic energy decays. The vorticity in the stagnation region is small. The effect of different values of vorticity on particle aggregation is different. Since the area with larger vorticity has greater energy exchange between fluid and particles, particle energy is pronouncedly decays, so the ring and primary deposits are formed, while the halo deposit is located in the area with small vorticity. It implies that these deposits are formed as a result of vortices formations.

### 5.2. The Effect of Flow Reynolds Number and Stokes Number on Deposits

**Figure 9:**
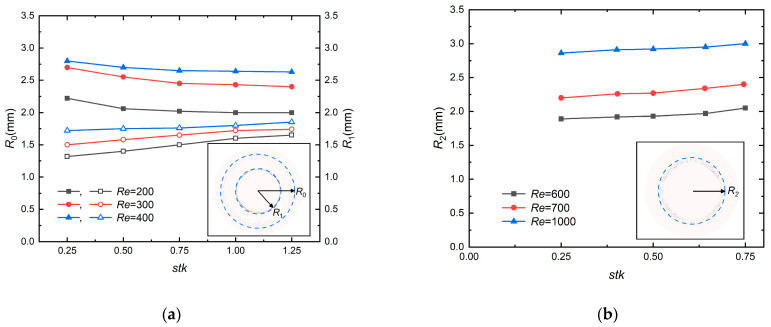
The radius of the (**a**) “halo” and (**b**) “ring” changes in terms of the different *Re* and *stk*. *R*_0_ and *R*_1_ are expressed as the radius of halo and primary deposit, respectively. *R*_2_ is expressed as the radius of ring. The solid points denote *R*_0_, and hollow points denote *R*_1_ in Figure 9a.

**Figure 10:**
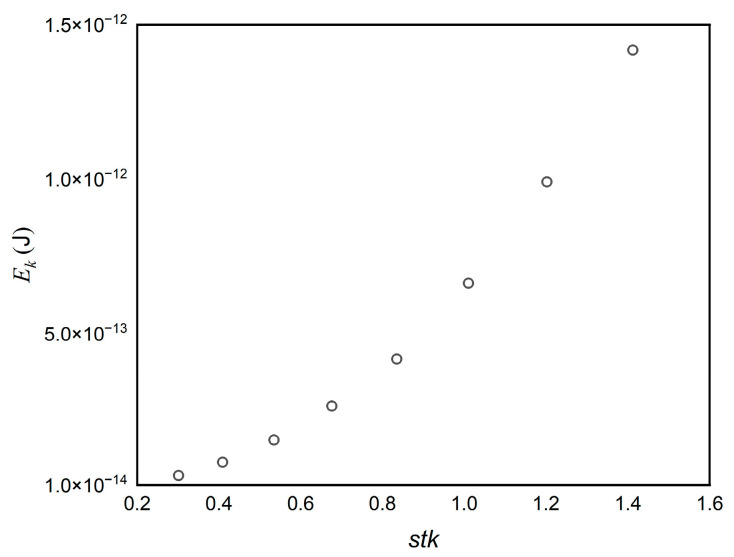
The *E_k_* of particles released at the same initial position for different *stk* in the same *y* coordinate in the upstream surface of the plate.

Figure 9 shows the radius of “halo” and “ring” changes in terms of the different *Re* and *stk* to understand the dependence of these factors on the dimensions of deposits. *R*_0_ and *R*_1_ are defined as the radius of the halo and primary deposit, respectively. The solid points denote *R*_0_, and hollow points denote *R*_1_ in Figure 9a. *R*_2_ is defined as the radius of the ring deposit. *stk* affects the deposit patterns in Figure 9, which was demonstrated before [5,6,7,10,41]. As *stk* increases, the halo gets closer to the primary deposit until it vanishes, thus *R*_0_ decreased, which agrees favorably well with the experimental result [8]. As *stk* increases, the value of *R*_0_ slightly decreases and tends to be stable as a consequence of the vortex, however, *R*_1_ gets larger when *stk* increases. To better explain this phenomenon, the kinetic energy (*E_k_*) of a particle released at the same initial position of the impactor inlet on the upstream surface of the plate is drawn in Figure 10 for different *stk* when *Re* = 200. *E_k_* increases with *stk*, which infers that the particle tends to drift at a higher velocity to the surface with the increasing *stk*. Consequently, particles roll further, and the radius of the primary deposit *R*_1_ is greater (Figure 9a). *R*_2_ varies similarly to *R*_1_ in Figure 9b. *Re* has a substantial effect on the radius of the halo and ring. As *Re* increases, the velocity of flow downstream of the nozzle increases and fewer particles are captured in the halo, due to the fact that particles have more kinetic energy with increasing fluid velocity, leading to the increase of *R*_0_, *R*_1_ and *R*_2_. Overall, the size and shape of these deposits vary greatly depending on the flow Reynolds number and Stokes number.

**Figure 11:**
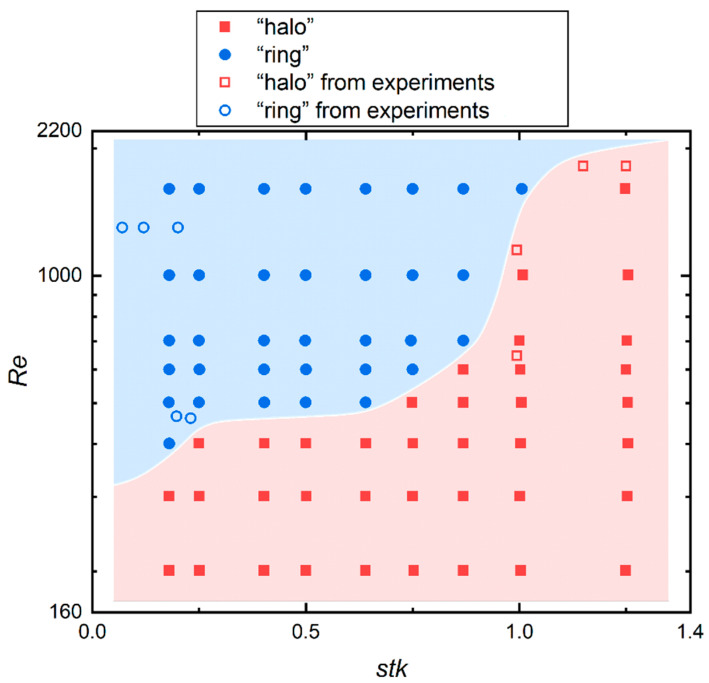
Categorization of the “halo” and “ring” deposit in terms of the value of *Re* and *stk*. The solid points and hollow points were taken from the simulation and experiment results [4,7,10,11,12], respectively.

Figure 11 shows the categorization of two particle deposition patterns (i.e., “halo” and “ring”) in terms of the value of *Re* and *stk*. The solid points and hollow points were taken from present simulations and experiment results from papers [4,7,10,11,12], respectively. A “halo” rarely occurs when *Re* is larger than 1000. The reason why a “halo” occurs with a bigger *stk* under the same *Re* has been explained above. However, for the same value of *stk*, a “halo” occurs with a smaller *Re*. This is because the particle size is larger for smaller *Re* under the same value of *stk*, thus the particle inertia plays a key role. The “halo” appears because the rebound phenomenon is more likely to occur for bigger particles. It can be supposed that the particle property is pronounced when the flow Reynolds number is relatively small. Another explanation is that *R*_0_ decreases whereas *R*_1_ increases with the increase of *Re* for the same value of *stk* in Figure 9a, so the halo and primary deposit get closer, forming the ring deposition. In summary, the different deposition patterns are the results of the combined action of the flow field and particles.

### 5.3. Energy Variation

**Figure 12:**
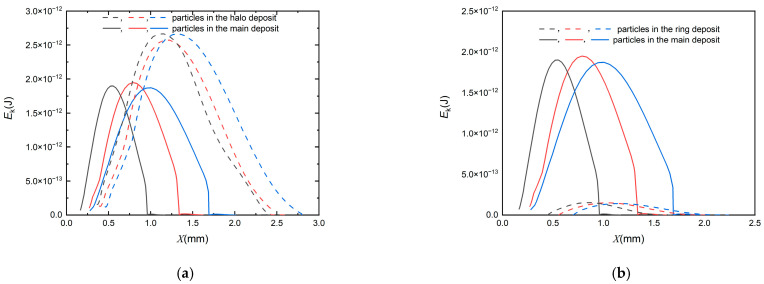
The kinetic energy of a particle changes with the particle location on the plate after the first particle–wall collision. (**a**) for “halo”. (**b**) for “halo” and “ring”.

To explain substantial reasons for the formation of two types of deposits, the following analysis is carried out from the perspective of energy. The potential energy of the particle can be ignored since it is extremely small in the present simulation during particle deposit. The initial kinetic energy of the particle is represented as $E_{ki}=\frac{1}{2}mv_{i}^{2}$. Its kinetic energy before impaction is sufficient to overcome the energy loss due to the particle–wall collision (*E_p-w_*), resulting in particle bounce, then the second particle–wall impingement occurs. The particle will roll after the impaction for a while in Figure 5. At this time, the particle is also subjected to the friction force and adhesion force from the plate surface whose energy conversion is collectively expressed as *E_d_*. This particle will stop until its energy dissipates. However, there is another situation, no rebound occurs in Figure 6, because particle kinetic energy is relatively small. To further analyze the deposition characteristics, Figure 12 shows kinetic energy of the particle changes with the particle location on the plate after the first wall–particle collision (several particles were selected for the statistical average method). Figure 12a illustrates *E_k_* of particles captured in the halo and primary deposits for *Re* = 700, *stk* = 1. The result demonstrates that the peak value of *E_k_* of particles captured in the halo deposition is generally higher than that of particles in the primary deposition as a consequence of the effect of stagnation region and energy loss caused by the rebound phenomenon mentioned in Figure 5. Figure 12b describes *E_k_* of particles in the “ring” and “halo” deposits for *stk* = 0.5 and *stk* = 1 when *Re* = 700, particles tend to rebound due to the increase of particle size leading to the increase of *E_k_*. As a result, a “halo” appears. There is a sharp decline in *E_k_* of particles in primary deposit when *E_k_* reduces from 5 × 10^−13^ to 0, approximately. When the kinetic energy of the particles drops to 0, the value of the corresponding abscissa is the *x* coordinate of the particle deposition. The results present that the *x* coordinate of halo deposit is larger than that of the primary deposit of “halo” in Figure 12a, while *x* coordinate of the ring deposit is larger than that of the primary deposit of “halo” in Figure 12b, which also corresponds to the location of the particle deposition mentioned before.

## 6. Conclusions

In this paper, the impingement flow characteristic and ultrafine particle deposition have been numerically demonstrated for the CFD-DEM method with particle–particle interaction, fluid–particle interaction, and particle–wall interaction concerned. Hertz–Mindlin model was chosen for particle–particle interaction, and Hertz–Mindlin with JKR model was selected for particle–wall interaction. Several conclusions can be drawn:

(1). The particle deposition pattern is determined by the flow structure of impinging jet and particle inertia. Two situations will occur (particle rebound or no rebound) after the particle impact on the plate, resulting in the formation of “halo” and “ring” deposits. Particles captured in the “halo” and “ring” show different energy variations during particle deposition. The ranges of variation of *E_k_* of particles captured in the “halo” deposit is generally larger than that of particles in the “ring” deposit for the same *Re*, and the peak value of *E_k_* of particles captured in the halo deposit is the highest.

(2). The particle deposition pattern under different *Re* and *stk* has been summarized, and categorization of the “halo” and “ring” deposition in terms of the value of *Re* and *stk* has been obtained. The “halo” deposit is easily formed with large *stk* and small *Re*, while the “ring” deposit is easily formed with small *stk* and large *Re*.

(3). *Re* has an influence on the vortical structures (size and location), further affecting magnitude of the particle deposit. These depositions are mostly located in the area with low fluid velocity. The ring and primary deposits are formed in the area with large vorticity, while the halo deposit is located in the area with small vorticity. In addition, the specific values of radius of these particle deposits for different *Re* and *stk* have been quantitatively given.

In summary, the CFD-DEM method has an advantage of particle concentration measurement. Therefore, it will be beneficial to control parameters to form the different particle deposition patterns, optimize the performance of the surface coating, and reduce error of particle size measurement. Future works will attempt to study the effects of temperature, particle shape and wall roughness on particle deposition.

## Figures and Tables

**Table 1:** Calculation conditions for CFD-DEM simulations.

Parameter Setting		Value	Unit
Fluid density		1.225	kg/m^3^
Particle density		1000	kg/m^3^
Wall density		7800	kg/m^3^
Particle size		2–13	µm
Coefficient of restitution	Particle–Particle	0.45	
	Particle–Wall	0.45	
Coefficient of static friction	Particle–Particle	0.5	
	Particle–Wall	0.8	
Coefficient of rolling friction	Particle–Particle	0.01	
	Particle–Wall	0.8	
Poisson’s ratio of particle		0.3	
Poisson’s ratio of wall		0.3	
Shear modulus of particle		1 × 10^7^	Pa
Shear modulus of wall		1 × 10^10^	Pa
Surface energy (Particle–Wall)		0.1	J/m^2^

**Table 2:** The test of grid independence in the flow field.

Case	Number of Grids	*u* (m/s)	Deviation from Experiment [34] (%)
Case 1	450,000	7.40	2.63
Case 2	550,000	7.48	1.58
Case 3	650,000	7.55	0.66
Case 4	750,000	7.58	0.26

**Table 3:** Comparisons of slope of the efficiency curves obtained from CFD-DPM method, CFD-DEM method, and the experimental result [34] in Figure 2.

Method	Slope of the Curve	Relative Error (%)
CFD-DPM	1.08	3.57
CFD-DEM	1.09	2.68
Experiment [34]	1.12	\

## Data Availability

Not applicable.

## Abbreviations

*ρ*	The gas density
*u*	The gas velocity
*p*	The gas pressure
*τ*	The viscous stress tensor of gas
*g*	The gravitational acceleration
*S_ai_*	The momentum transfer term between the fluid and the *i*th particle phase
*F*_*a*,*i*_	The total force exerted on the *i*th particle
*V_cell_*	The volume of cell
*Re*	The flow Reynolds number
*m_p_*	The particle mass
*u_p_*	The particle velocity
*u_py_*	The velocity magnitude of particles in the *y* direction
*F_D_*	The drag force
*I_p_*	The rotational inertia of the particle
*ω_p_*	The angular velocity of the particle
*r_c_*	The distance from the contact point to particle center
*µ*	The fluid dynamic viscosity
*ρ_p_*	The particle density
*d_p_*	The particle size
*Re_p_*	The particle Reynolds number
*C_D_*	The drag coefficient
*stk*	The Stokes number
*C_c_*	The slip correction factor
*E_k_*	The kinetic energy of a particle
*E_p-w_*	The particle–wall collision
*E_d_*	The friction force and adhesion force
*F_c_*	The contact force acted on the particle
*F_ct_*	The tangential component of the contact force
*F_cn_*	The normal component of the contact force
*F_et_*	The tangential elastic force
*F_dt_*	The tangential damping force
*F_en_*	The normal elastic force
*F_dn_*	The normal damping force
*F_JKR_*	The adhesion force
*γ*	The surface energy
$E^{\prime }$	The equivalent Young’s modulus
$R^{\prime }$	The equivalent radius of the particle
*a*	The contact half-width
*R*	The particle radius
*E*	The Young’s modulus
ν	The Poisson’s ratio
*δ_n_*	The normal overlap
*W*	The round–nozzle diameter
*T*	The length of the nozzle throat
*S*	The distance from the nozzle to the impaction plate
*D*	The diameter of the impaction plate
*R*_0_	The radius of the halo deposit
*R*_1_	The radius of the primary deposit
*R*_2_	The radius of the ring deposit
*X*	The *x* coordinate of the particle deposition
